# Prevalence of and Risk Factors for Peripheral Neuropathy in Chinese Patients With Diabetes: A Multicenter Cross-Sectional Study

**DOI:** 10.3389/fendo.2018.00617

**Published:** 2018-11-05

**Authors:** Qi Pan, Quanmin Li, Wei Deng, Dong Zhao, Lin Qi, Wei Huang, Li Ma, Hongmei Li, Yufeng Li, Xiaofeng Lyu, Aihong Wang, Hebin Yao, Xiaoyan Xing, Lixin Guo

**Affiliations:** ^1^Department of Endocrinology, Beijing Hospital, National Center of Gerontology, Beijing, China; ^2^Department of Endocrinology, General Hospital of the PLA Rocket Force, Beijing, China; ^3^Department of Endocrinology, Beijing Jishuitan Hospital, Beijing, China; ^4^Department of Endocrinology, Beijing Luhe Hospital, Capital Medical University, Beijing, China; ^5^Department of Endocrinology, Beijing Yanhua Hospital, Beijing, China; ^6^Department of Endocrinology, Beijing Haidian Hospital, Beijing, China; ^7^South Section, Department of Endocrinology, Guang'anmen Hospital, China Academy of Chinese Medical Sciences, Beijing, China; ^8^Department of Endocrinology, China Meitan General Hospital, Beijing, China; ^9^Department of Endocrinology, Beijing Pinggu Hospital, Beijing, China; ^10^Department of Endocrinology, Army General Hospital of PLA, Beijing, China; ^11^Department of Endocrinology, The 306th Hospital of PLA, Beijing, China; ^12^Department of Endocrinology, Navy General Hospital, Beijing, China; ^13^Department of Endocrinology, China-Japan Friendship Hospital, Beijing, China

**Keywords:** peripheral neuropathy, diabetic, a retrospective multicenter study, diagnostic criteria, risk factors

## Abstract

Diabetic peripheral neuropathy (DPN) is the most common complication of diabetes, and its progression significantly worsens the patient's quality of life. This study investigated the prevalence and risk factors associated with DPN in a large sample of Beijing individuals with type 1 and 2 diabetes, as well as compared the diagnostic methods for DPN. A total of 2,048 diabetic patients from 13 centers in Beijing were assessed for DPN through questionnaires and examination. Patients were divided into DPN group and suspected DPN/non-DPN group. The demographic, clinical and biological characteristics between the two groups were compared. Binary logistic regression analysis was performed to identify potential variables associated with DPN in diabetic patients. The diagnostic methods for DPN were also compared. Among the 2,048 diabetic patients, 73 cases of type 1 diabetes mellitus, 1,975 cases of type 2 diabetes were included in this study. Among them, 714 (34.86%) were identified with DPN, 537 (26.22%) were suspected of having DPN, and 797 (38.92%) were identified without DPN. Patient's age, duration of diabetes, and diabetic retinopathy were the significant independent risk factor for DPN among patients with type 2 diabetes. The odds ratio (OR) was 1.439 (95% confidence interval (CI): 1.282–1.616, *P* < 0.001), 1.297 (95% CI: 1.151–1.462, *P* < 0.001), and 0.637 (95% CI: 0.506–0.802, *P* < 0.001), respectively. Ankle reflex, temperature sensation plus vibration sensation are the best screening test for patients with type 1 and 2 diabetes. The Youden indexes were 62.2 and 69.8%, respectively. The prevalence rates of DPN in the Chinese patients with type 1 and type 2 diabetes in Beijing were 21.92 and 35.34%, respectively. Patient's age, duration of diabetes, and diabetic retinopathy are the independent risk factors for DPN.

## Introduction

The prevalence of diabetes is increasing globally, particularly in developing countries. An estimated 20.8 million people living in China have diabetes in 2000, and this number will rise to 42.3 million by 2030 (1). Neuropathy is considered one of the most common microvascular complications associated with both types of diabetes, in which diabetic peripheral neuropathy (DPN) is the most common type (2). DPN has been defined by international consensus guidelines as the presence of symptoms and/or signs of peripheral nerve dysfunction in people with diabetes after the exclusion of other causes (3). DPN is characterized by progressive, distal-to-proximal degeneration of peripheral nerves that leads to a wide range of neuropathic symptoms, such as numbness, burning, prickling/tingling, sharp pains or cramps, extreme sensitivity to touch, allodynia and loss of balance and coordination (4, 5). The prevalence of DPN among individuals with diabetes is reported to be as high as 50% (6), but a large variation in prevalence rate exists across studies, which may be due to the different patient populations and the lack of consensus on its diagnostic criteria (7). DPN has been shown to be a significant independent risk factor for diabetic foot, which is a leading cause of foot ulceration and subsequent lower limb amputations in patients with diabetes, and associated with premature mortality (8).

Early detection and good glycemic control can delay or prevent adverse outcomes resulting from DPN, thereby improving patients' quality of life. Before 2010, there was no clear diagnostic criterion for DPN, which is mainly a diagnosis of exclusion. After 2010, an active diagnosis based on symptoms, signs and neurological functions was recommended, which is more stringent diagnostic criteria (9). The 2013 Chinese guidelines for the prevention and treatment of type 2 diabetes mellitus suggest repeat screening for DPN at least once per year following the diagnosis of diabetes (10).

Since more than 50% of DPN patients are found to be asymptomatic, and endocrinologists do not pay attention to or are not familiar with the clinical screening method for DPN, the screening rate is still low in China. To our knowledge, there were limited published data regarding the prevalence of DPN in Beijing. On the basis of the National DPN Screening Demonstration Project, Chinese Diabetes Society (CDS) Beijing Branch developed a survey to explore the prevalence of DPN in diabetic patients living in Beijing. In the present study, we aim to estimate the prevalence and risk factors associated with DPN in a large sample of Beijing individuals with type 1 and 2 diabetes, which may lead to improved preventive measures and care for diabetic patients.

## Materials and methods

### Subjects

A total of 2,048 outpatients were enrolled by the random sampling from 13 hospitals in Beijing, including 193 from Beijing Hospital, 161 from Beijing Jishuitan Hospital, 160 from China-Japan Friendship Hospital, 165 from Beijing Haidian Hospital, 158 from Army General Hospital of PLA, 200 from South Section of Guang'anmen Hospital, 98 from Navy General Hospital, 85 from The 306th Hospital of PLA, 157 from Beijing Luhe Hospital, 160 from China Meitan General Hospital, 160 from Beijing Pinggu Hospital, 200 from Beijing Yanhua Hospital, and 151 from PLA Rocket Force General Hospital. Men and women with type 1 or type 2 diabetes mellitus were eligible to participate if they were conscious, had no language communication barriers, and could actively cooperate with the completion of the evaluation. Diagnosis of diabetes was made according to 1999 World Health Organization (WHO) criteria (11).

The exclusion criteria were as follows: (1) subjects who had neuropathy resulting from other causes, such as cervical and lumbar spine lesions (nerve root compression, spinal canal stenosis or cervical and lumbar degeneration), cerebral infarction and Guillain-Barré syndrome (GBS); (2) subjects who had arteriovenous vascular disease (venous thrombosis or lymphangitis); (3) subjects who had neurotoxicity produced by chemotherapy drugs and nerve damage caused by metabolic toxins due to renal insufficiency; (4) pregnant or lactating women; (5) subjects who suffered from mental illness; and (6) subjects who did not cooperate or was considered unfit for the study. 50 subjects were excluded from the study, where 36 had neuropathy, 2 were pregnant, 7 suffered from mental illness and 5 were considered unsafe for the study. Age, sex, lifestyle, duration, complications, medication, etc. from all the participants who met the inclusion and exclusion criteria were collected. The study was approved by the institutional review board(s) at each study site. Informed consent was obtained from all participants before the survey.

### Diagnostic criteria for DPN

The diagnostic criteria for DPN was based on the 2017 diagnostic methods proposed by the American Diabetes Association (12) and included all of the following: (1) a definitive history of diabetes; (2) the presence of neuropathy at or after diagnosis of diabetes; (3) clinical symptoms and signs consistent with DPN; (4) the presence of clinical symptoms (pain, numbness, paresthesia, etc.) along with one anomaly of the five examinations (ankle reflex, acupuncture pain, vibration sensation, temperature sensation and pressure sensation), or the absence of clinical symptoms along with two anomalies of the five examinations. The clinical diagnosis of DPN was mainly based on clinical symptoms, such as pain, numbness, paresthesia, etc. Suspected DPN was present if the participant had symptoms of DPN but no signs, or no symptoms of DPN but one or more positive signs.

The five examinations were performed at room temperature of 25°C. The procedures of the examinations are listed below. The investigators were trained and used the same instructions for the examinations.

1. Ankle reflex. The patient sits on a chair and both ankles are relaxed. The foot is gently supported, and the Achilles tendon is stroke using a rubber hammer. Positivity is defined as absent or reduced ankle reflex in both feet.
2. Acupuncture pain. The arm skin is gently pricked using a pin to give the patient a reference sensation of pain. The patient closes both eyes. Then both feet are pricked once using the pin. No feeling of pain in either side of the foot is a positive result.
3. Vibration sensation. A 128 Hz tuning fork is placed onto the wrist or elbow to give the patient a reference feeling of vibration or non-vibration. The patient closes both eyes. Then a vibrating tuning fork is placed onto the dorsal surface of the metatarsal joint of the great toe. The patient is asked to answer if he/she is feeling vibration. The test is repeated three times for both sides. Positivity is defined as two or three wrong answers for either side.
4. Temperature sensation. A Tip Therm is placed onto the foot skin with its metal side (cooler) and polymer side (warmer). Positivity is defined as inability to differentiate the two sides of the Tip Therm in either side of the foot.
5. Pressure sensation. A 10-g monofilament is gently bent for 1–2 s with one side pushed upon the arm skin to give the patient a reference feeling of pressure sensation. The patient closes both eyes. For each foot, the pressure sensation is tested at the plantar surface of the great toe, the lateral side of the anterior sole, and medial side of the anterior sole. Positivity is defined as lost of pressure sensation in any tested sites of either foot.

### Statistical analysis

Continuous variables distributed normally were expressed as a mean ± standard deviation (SD), and were compared between two groups by the independent sample *t*-test. Non-normally distributed variables were presented as medians (interquartile range), and were compared between two groups by the Mann-Whitney U test. Categorical variables were reported as frequencies and proportions, and were compared between two groups using the Chi-Square test. Binary logistic regression analysis was conducted to identify potential variables associated with DPN in diabetic patients, and was adjusted for age and gender. We used an acceptable working curve to evaluate the sensitivity and specificity of different methods for diagnosing DPN. The statistical analyses were performed using the software SPSS (version 19.0; SPSS Inc., Chicago, IL, USA) and MedCalc 17.6. *P* < 0.05 was considered statistically significant.

## Results

### Baseline characteristics of the study population

The characteristics of the study population are depicted in Table 1. A total of 2,048 patients with diabetes (73 with type 1 and 1,975 with type 2 diabetes) from 13 centers participated in the present study and have completed data for the variables of interest. In the diabetes patients, 714 (34.86%) were identified with DPN, 537 (26.22%) were suspected of having DPN, and 797 (38.92%) were not having DPN. Patients were divided into 3 groups: DPN group, suspected DPN group, and non-DPN group. The demographic, clinical and biological characteristics of three groups are presented in Table 1. Among patients with type 1 and 2 diabetes, those with DPN have an older age and longer diabetes duration compared to those with suspected group and without DPN group (Type 1 age: 57.19 ± 11.07 vs. 56.21 ± 12.37, 48.79 ± 13.88, *P* = 0.045; Type 2: 62.22 ± 9.55 vs. 59.30 ± 10.54, 56.01 ± 11.34, *P* < 0.001; Duration: >10 vs. 5–10 and < 4 years, P1 = 0.031, P2 < 0.001). In the type of 1 and 2 diabetes, DPN patients have high concentration of HbA1c than suspected DPN group and non-DPN group (HbA1c: 9.725 ± 1.70 vs. 7.99 ± 1.53 and 7.03 ± 1.24). In metformin use and metformin duration, type 1 and 2 diabetes patients present differences between three groups (All *P*-values are < 0.05). Among patients with type 2 diabetes, there are differences between the three groups in hypoglycemia, hypertension, metformin dose, diabetic retinopathy and diabetic neuropathy. All *P*-values are < 0.05. No significant difference was found in the other characteristics between three groups.

**Table 1:** Demographic, clinical, and biological characteristics in patients with diabetes.

**Characteristic**	**Type 1 diabetes**			***P*-value**	**Type 2 diabetes**			***P*-value**
	**DPN (*n* = 16)**	**Suspected DPN (*n* = 29)**	**Non-DPN (*n* = 28)**		**DPN (*n* = 698)**	**Suspected DPN (*n* = 508)**	**Non-DPN (*n* = 769)**	
Age, years	57.19 ± 11.07	56.21 ± 12.37	48.79 ± 13.88	0.045	62.22 ± 9.55	59.30 ± 10.54	56.01 ± 11.34	< 0.001
Male, *n* (%)	9 (56.25)	12 (41.38)	18 (64.29)	0.215	334 (47.85)	251 (49.41)	408 (53.06)	0.124
BMI, kg/m^2^	–	–	–	0.945	–	–	–	0.267
< 24	6 (37.50)	13 (44.83)	10 (35.71)	–	247 (35.39)	178 (35.04)	237 (30.82)	–
24–27.9	8 (50.00)	12 (41.38)	13 (46.43)	–	315 (45.13)	219 (43.11)	366 (47.59)	–
≥ 28	2 (12.50)	4 (13.79)	5 (17.86)	–	136 (19.48)	111 (21.85)	166 (21.59)	–
Smoking, *n* (%)	5 (31.25)	4 (13.79)	11 (39.29)	0.090	174 (24.93)	130 (25.64)	200 (26.01)	0.892
Drinking, *n* (%)	6 (37.50)	5 (17.24)	8 (28.57)	0.309	159 (22.78)	125 (24.61)	197 (25.62)	0.444
Diabetes duration, years	–	–	–	0.031	–	–	–	< 0.001
< 4	2 (12.50)	7 (24.14)	14 (50.00)	–	150 (21.49)	147 (28.94)	301 (39.14)	–
5–10	2 (12.50)	6 (20.39)	6 (21.43)	–	128 (18.34)	102 (20.08)	185 (24.06)	–
> 10	12 (75.00)	16 (55.17)	8 (28.57)	–	420 (60.17)	259 (50.98)	283 (36.80)	–
Cr, μmol/L	71.0 (44.0–107.0)	82.0 (53.0–111.0)	0 (0–0)	0.699	62.0 (51.0–76.6)	61.6 (51.6–73.3)	64.1 (54.9–76.2)	0.052
UAER, μg/min	69.7 (12.5–254.5)	27.4 (21.0–33.8)	350 (350–350)	0.601	16.0 (9.3–43.7)	16 (5.6–79.5)	17.0 (9.2–44.0)	0.930
HbA1c, %	9.72 ± 1.70	7.99 ± 1.53	7.03 ± 1.24	< 0.001	8.47 ± 1.83	7.65 ± 1.53	7.44 ± 1.61	< 0.001
Hypoglycemia, *n* (%)	7 (43.75)	8 (27.59)	8 (28.57)	0.489	218 (31.23)	139 (27.36)	159 (20.68)	< 0.001
Hypertension, *n* (%)	8 (50.00)	11 (37.93)	13 (46.43)	0.693	352 (53.09)	238 (47.89)	340 (44.44)	0.005
On metformin, *n* (%)	11 (68.75)	22 (75.86)	7 (25.00)	< 0.001	528 (75.64)	351 (69.09)	310 (40.31)	< 0.001
Insulin	3 (15.79)	2 (20)	8 (28.57)	0.575	144 (26.04)	94 (20.52)	126 (21.65)	0.079
Acarbose	5 (26.32)	2 (20)	8 (28.57)	0.869	144 (26.04)	122 (26.64)	148 (25.43)	0.906
Glucophage	3 (15.79)	1 (10)	1 (3.57)	0.344	54 (9.76)	58 (12.66)	31 (5.33)	< 0.001
Metformin dose, mg	1.54 ± 0.80	1.42 ± 0.43	0.92 ± 0.74	0.058	1.54 ± 0.82	1.35 ± 0.70	0.92 ± 0.86	< 0.001
Duration of metformin use, months	120 (60–156)	60 (12–120)	12 (6–20)	0.016	96 (36–136)	36 (12–86)	12 (0–36)	< 0.001
Diabetic retinopathy, *n* (%)	3 (18.75)	5 (17.24)	3 (10.71)	0.708	199 (28.59)	106 (20.87)	106 (13.80)	< 0.001
Diabetic neuropathy, *n* (%)	6 (37.50)	7 (24.14)	0 (0.00)	0.004	149 (21.38)	58 (11.44)	39 (5.07)	< 0.001

*Data are mean ± SD, %, or median (interquartile range)*.

*BMI, body mass index; HbA1c, glycosylated hemoglobin; Cr, serum creatinine; UAER, urinary albumin excretion rates; TG, triglyceride; TC, total cholesterol; LDL, low density lipoprotein; HDL, high density lipoprotein*.

### Risk factors of DPN

We performed regression analysis with the variables which show significant difference among the three groups to determine the risk factors for DPN. In logistic regression analysis, gender and age were adjusted, and the results suggested diabetes duration and diabetic retinopathy were the significant independent risk factors for DPN among patients with type 2 diabetes. The odds ratios (ORs) were 1.297 (95% confidence interval (CI): 1.151–1.462, *P* < 0.001) and 0.637 (95% CI: 0.506–0.802, *P* < 0.001), respectively (Table 2).

**Table 2:** Adjusted OR and 95% CI for factors associated with DPN.

**Variable**	**Type 1 diabetes**		**Type 2 diabetes**	
	**OR (95% CI)**	***P*-value**	**OR (95% CI)**	***P*-value**
Age^*^	1.240 (0.677–2.257)	0.490	1.439 (1.282–1.616)	< 0.001
Gender (male vs. female)	0.971 (0.299–3.153)	0.961	1.055 (0.871–1.278)	0.586
Diabetes duration^*^	2.241 (0.997–5.035)	0.051	1.297 (1.151–1.462)	< 0.001
Diabetic nephropathy (yes vs. no)	–	–	0.795 (0.564–1.121)	0.191
Diabetic retinopathy (yes vs. no)	–	–	0.637 (0.506–0.802)	< 0.001

* *As a continuous variable*.

### Comparison of diagnostic methods for DPN

Different diagnostic methods and their combinations used for screening DPN in patients with type 1 and 2 diabetes were summarized in Tables 3, 4. For patients with both type 1 and 2 diabetes, ankle reflex, temperature sensation plus vibration sensation yielded the highest sensitivity of 93.70 and 94.14% for screening DPN, however, the Youden index were only 62.2 and 69.8%.

**Table 3:** Patients with positive results of the neurological examinations.

	**Type 1 diabetes**			***P*-value**	**Type 2 diabetes**			***P*-value**
	**DPN (*n* = 16)**	**Suspected DPN (*n* = 29)**	**Non-DPN (*n* = 28)**		**DPN (*n* = 698)**	**Suspected DPN (*n* = 508)**	**Non-DPN (*n* = 769)**	
Ankle reflex	10 (62.5)	15 (51.7)	1 (3.6)	< 0.001	385 (55.2)	150 (29.5)	16 (2.1)	–
Acupuncture pain	0	0	0	–	84 (12.0)	16 (3.1)	4 (0.5)	< 0.001
Temperature sensation	7 (43.8)	2 (6.9)	0 (0.0)	< 0.001	354 (50.7)	84 (16.5)	15 (2.0)	< 0.001
Vibration sensation	6 (37.5)	1 (3.4)	1 (3.6)	0.001	312 (44.7)	53 (10.4)	15 (2.0)	< 0.001
Pressure sensation	4 (25.0)	2 (6.9)	1 (3.6)	0.055	190 (27.2)	31 (6.1)	5 (0.7)	< 0.001

**Table 4:** Comparison of diagnostic methods for DPN in patients with type 1 and 2 diabetes.

	**Type 1 diabetes**			**Type 2 diabetes**		
	**Sensitivity (%)**	**Specificity (%)**	**Youden index (%)**	**Sensitivity (%)**	**Specificity (%)**	**Youden index (%)**
Ankle reflex	62.5	71.5	34.4	55.2	87.2	42.4
Acupuncture pain	–	–	–	12.0	98.7	10.7
Temperature sensation	43.7	96.5	40.2	50.7	92.5	43.2
Vibration sensation	37.5	96.5	34.0	44.7	94.7	39.4
Pressure sensation	25.0	94.7	19.7	27.2	97.3	24.5
Ankle reflex/temperature sensation	81.2	68.4	49.7	83.5	79.9	63.4
Ankle reflex/vibration sensation	81.2	70.2	51.4	78.1	82.3	60.4
Temperature sensation/vibration sensation	62.5	94.7	57.2	73.8	87.5	61.3
Ankle reflex/temperature sensation/vibration sensation	93.7	68.4	62.2	94.1	75.6	69.8

## Discussion

DPN is significantly associated with a high morbidity and its progression that causes great physical and mental sufferings to the patients and their families, worsens the quality of and life span of the patients. DPN also substantially increases diabetes-related health costs (13). DPN can predict foot ulcer, lower-extremity amputation and mortality in diabetic patients (8). There is a need for a better understanding of the prevalence of DPN and risk factors associated with DPN to further develop the preventive strategies for DPN in patients diagnosed with diabetes in China. This study was a multi-center survey of 2,048 cases in 13 hospitals of Beijing.

The diagnosis rate for DPN via questionnaire is generally low, and early screening could increase the diagnosis rate. The prevalence of DPN in this multicenter cohort of patients with type 1 and type 2 diabetes was 21.92 and 35.34%, respectively. This prevalence is high, and if DPN screening was not performed in time, the chance to diagnose early DPN would be missed. Thus, diabetic patients should be screened for DPN even if they are not experiencing neuropathic symptoms. Several studies have described the prevalence of DPN in China in specific settings or sub-populations. Estimated prevalence rates among individuals with type 2 diabetes range from 8.4 to 61.8% (14–17). A study conducted by Lu B et al. investigated the prevalence and risk factors associated with DPN in type 2 diabetic patients aged over 30 in the Shanghai downtown, and estimated the prevalence of DPN to be as high as 61.8% (15). In another study by Lu B et al., the prevalence of DPN was 8.4% in subjects with diabetes mellitus in a community-based Chinese population in Shanghai (16). A cross-sectional study in a type 2 diabetic cohort from a community-based Beijing's population estimated a DPN prevalence of 54.5% (17). A randomized, multi-center epidemiological study of 12 sites in 8 Chinese cities estimated a DPN prevalence of 17.02% in the total population, 18.28% in known cases of diabetes and 6.35% in newly diagnosed cases of diabetes, and this study also reported a large variation of DPN prevalence among research centers (14). The prevalence of DPN in different countries was also varied. In the Sudanese cohort of 424 individuals with type 2 diabetes, a prevalence of 68.2% was reported (18). The prevalence of DPN in this population was 19.9% in a Saudi Arabic population (19). The proportions of diabetes patients diagnosed with DPN were 9.6, 10.7, 14.1, and 23.1% for Spain, France, the UK and Italy (4). Estimated prevalence rate of DPN among French adults with diabetes was 11% (7). This large variation of DPN prevalence may be attributed to different types of diabetes, study design, different methods of patient selection, individual differences, discrepancies in diabetes duration, differences in the diagnostic criteria employed (e.g., If NCV are available, some patients with suspected DPN may be converted to those with confirmed DPN), race and the year when a study was conducted. The lack of international consensus on the diagnostic criteria of DPN also is an important reason of the large variation in DPN prevalence. Investigators may use very different DPN diagnostic criteria in these studies.

The mechanisms underlying DPN pathogenesis are not fully understood. Mitochondrial dysfunction, oxidative stress, inflammation and altered gene expression may be involved (20). Hyperglycemia is a major pathophysiologic factor in the development of DPN in diabetes. Strict glucose control is fundamental for long-term prevention and management of DPN (21). It has been shown that poor glycemic control contributes to the development of DPN (22). However, our study did not find that poor glycemic control was a risk factor of DPN. The possible reason might be that most of patients were Beijing residents who enjoyed a well-established medical system and well-funded governmental medical insurance. In addition, most of our patients had type 2 diabetes and their glucose levels were well-managed in a relatively narrow range.

Among our patients with both types of diabetes, those with DPN had a longer duration of diabetes compared to those with suspected or without DPN. Logistic regression analysis further found a statistically significant relationship between DPN and duration of diabetes after controlling for age and gender. It implied that diabetes duration and diabetic retinopathy was an independent risk factor for DPN. Previous studies have also confirmed a significant association between longer duration of diabetes and DPN in diabetic subjects (2, 14, 18, 23). Even though not a modifiable risk factor, duration of diabetes is particularly important for early identification and management of DPN.

We also observed that the prevalence rates of diabetic nephropathy were significantly higher in type 2 diabetic subjects with DPN, but our regression analysis found no association between DPN and diabetic nephropathy in this population. Previous studies have suggested that age (14, 23, 24), elevated level of HbA1c can predict risk of DPN (14, 23, 25), and our study received the same result as the studies. In addition, as the use and dosage of metformin have an impact on the absorption of vitamin B12, leading to the occurrence of DPN, the patients with long-term use of high-dose metformin should be taken more care.

An effective screening method is also required to diagnose DPN early in high risk population to prevent future foot ulcers and amputation. The complex and extensive clinical presentation of DPN and the lack of the objective evaluation indexes make quantitative diagnosis and screening quite difficult. The diagnosis of a DPN is most often made on clinical grounds with a suggestive clinical history and neurologic examination. In our study, sign screening was used for DPN detection, which did not rely on machines and professional inspectors. It is suitable for promotion at the grassroots level. In this study, ankle reflex, temperature sensation plus vibration sensation is the best screening test for DPN patients with type 1 and 2 diabetes.

Our study has limitations. First, nerve conduction study was not performed, which may result in missed diagnosis. Second, the laboratory results were retrospectively collected. Third, the complications were investigated through patient complaints and medical records.

In conclusion, this multi-center study shows that the prevalence rates of DPN in the Chinese patients with type 1 and type 2 diabetes were 21.92 and 35.34%, respectively. Patient's age, duration of diabetes, and diabetic retinopathy are independent risk factors for DPN.

## Author contributions

QP, QL, WD, and DZ designed the study, conducted the literature research. LQ, WH, LM, and HL carried out the data interpretation, and drafted the manuscript. YL, XL, AW, and HY carried out data acquisition and drafted part of the manuscript. LG and XX contributed in interpretation of the data and significantly improved the manuscript. All authors read and approved the final manuscript.

### Conflict of interest statement

The authors declare that the research was conducted in the absence of any commercial or financial relationships that could be construed as a potential conflict of interest.
